# Translation of the focus toward excellence in translational science: comment on “TDP-43 Repression of Nonconserved Cryptic Exons is Compromised in ALS-FTD”

**DOI:** 10.3325/cmj.2015.56.493

**Published:** 2015-10

**Authors:** Roland Pochet, Charles Nicaise, Dinko Mitrečić

**Affiliations:** 1 Histology, Neuroanatomy and Neuropathology Faculty of Medicine, Université Libre de Bruxelles, Brussels, Belgium; 2 Laboratory for Stem Cells, Croatian Institute for Brain Research, University of Zagreb School of Medicine, Zagreb, Croatia; 3 Laboratory Neurodegeneration and Regeneration, URPHYM-NARILIS, Université de Namur, Namur, Belgium

In the past weeks, a lot of attention has been directed to the article by Philip Wong and colleagues – TDP-43 Repression of Nonconserved Cryptic Exons Is Compromised in ALS-FTD published in *Science* on August 7 (1). Several internet portals claimed that this study discovered the cause of amyotrophic lateral sclerosis (ALS) (2), provoking a number of reactions from the public, still sensibilized to ALS as a result of a recent world-wide fundraising event – the Ice Bucket Challenge. Some patients’ associations even organized celebration parties with the message that “the Ice Bucket Challenge enabled scientists to finally solve the ALS mystery.” This was also supported by Wong and colleagues, who expressed gratitude for donations and invited everyone to continue to raise the budget in this way (3).

## ALS and the Ice Bucket Challenge

Although the Ice Bucket Challenge raised 220 million USD worldwide it should be stressed that this amount constitutes a small portion of the budget continuously injected into ALS research. The Ice Bucket Challenge has more an empathic than a scientific effect, resulting in an interesting social phenomenon. Similar phenomenon occurred one year ago when some patients’ organizations announced that ALS can be cured in China (4). Without trying to analyze why ALS, as only one of numerous fatal human diseases, is surrounded by such hype, we would like to discuss the importance of the publication by Wong and colleagues.

## What was indeed discovered by Wong and colleagues?

For more than a decade, it has been known that one of the hallmarks of ALS is accumulation of protein TDP-43. However, its link to dying of motor neurons has remained a mystery. Wong and colleagues suppressed the normal function of protein TDP-43 and observed a surprising result: appearance of abnormal strands of RNA. Indeed, when they compared brains of patients affected by ALS with control patients, it become obvious that the lack of TDP43 allowed the production of cryptic RNA, which normally should not be present. Obviously, the same phenomenon occurs when TDP3 is not deleted, but is abnormally accumulated within the cells. The most valuable finding of this publication is that the authors prevented the occurrence of these RNA strands by a genetic engineering approach that imitated the activity of wild type TDP43 and thus prevented cells from dying.

This important finding did not come out of nowhere. It was achieved thanks to the gradual progress in understanding of RNA-binding proteins data accumulated in the last two decades. Already in 1992, the concept of “potential for cryptic splice site usage” was introduced, which allowed scientists to predict the relative proportion of exon skipping vs cryptic splice site (5). In 2009, it was further demonstrated that TDP-43 exhibited a high affinity in binding to UG-rich RNA (6). It is important to notice that TDP-43 binds to more than 6000 RNA targets in the brain, roughly 30% of the total transcriptome (7). Structural TDP-43 analysis has shown that, besides containing two RNA recognition motifs followed by a prion-like domain, which is essential in determining aggregation, aggregates are ubiquitinated and hyperphosphorylated, which is a hallmark of all neurodegenerative diseases (8).

However, the intention of this study was not to discover either the cause of TDP43 accumulation or the link to selective death of motoric neurons. In addition, their finding that the exons suppressed by TDP43 are not conserved, ie, that they differ in humans and mice, has a serious consequence on the applicability of mouse models for ALS research (1). We are still far away from understanding the cause of ALS, and it is highly questionable whether we will ever find a unique cause of all its forms. Since its seminal description in the 19th century, ALS has been defined and treated as a single neurodegenerative disorder despite broad clinical heterogeneity. Nowadays, it is more and more recognized as a heterogeneous syndrome with diverse genetic causes and pathology background. Genetic studies have identified abnormal repeat expansions in *C9ORF72*, or mutations in *SOD1*, *TDP43*, *FUS* genes responsible for most of familial forms of ALS. Genetic variants in genes known to cause familial ALS were found in a small fraction of sporadic patients, leaving the etiology of the sporadic form largely unknown (9). The genetic heterogeneity combined with environmental factors or patient-specific history might explain the clinical heterogeneity. Why is Stephen Hawking still alive with ALS when so many other patients are expected to die 2 to 5 years after diagnosis? To add to this clinical complexity, ALS often overlaps with progressive muscular atrophy, primary lateral sclerosis, frontotemporal dementia, which makes the identification of a unique therapeutic target challenging (9). Is ALS a single disease that triggers a single common downstream event (ultimate death of upper and lower motor neurons) with many different upstream events (RNA processing defect, protein misfolding, toxic gain-of-function, protein loss-of-function) or a clinical syndrome encompassing multiple subtypes of diseases with independent etiology and pathogenic mechanisms?

These considerations emphasize the need to innovate and to adopt other approaches; apart from the strategy based on stem cells, which raised the highest interest in the last decade (10), we need approaches that take into account the genetic background of ALS patients. As ALS patient brain tissue remains largely inaccessible, induced pluripotent stem cell (IPSC) technology offers the opportunity to generate patient-specific disease models in-a-dish, even when the genetic risk factors are unidentified. Motor neurons or glial cells derived from these IPSCs might help to identify different ALS phenotypes and develop targeted therapies for each subtype (11). Through groundbreaking advances in the genetic of ALS and new tools allowing personalized treatment, we are about to forget a two-century old paradigm of treating all ALS patients in an identical way. Another concept which emerges as unavoidable is the increased use of modern neuroimaging methods that now allow detecting the presence of a target molecule in a single cell in a living experimental animal or patient.

In conclusion, although the study by Wong and colleagues represents a gem of translational science, its greatest value is in showing us the direction in which we need to go. It can be expected that discovering the chain of events that trigger the disease onset on the molecular level will finally allow, combined with precise molecular visualization, the design the next generation of molecules with a potent and personalized therapeutic effect.
